# Evaluation of serum procalcitonin level as a biomarker for disease severity in COVID-19 patients

**DOI:** 10.1016/j.nmni.2021.100922

**Published:** 2021-07-24

**Authors:** A. Waris, M. Din, N. Iqbal, L. Yar, A. Khalid, M. Nawaz, A. Baset, M. Ali

**Affiliations:** 1) Department of Biotechnology, Quaid-i-Azam University, Islamabad, Pakistan; 2) Department of Statistics, Quaid-i-Azam University, Islamabad, Pakistan; 3) Sahiwal Medical College, University of Health Sciences, Lahore, Pakistan; 4) Department of Zoology, Bacha Khan University, Charsadda, Pakistan

**Keywords:** Bacterial co-infection, biomarker, -COVID 19, disease severity, procalcitonin

## Abstract

The pandemic of coronavirus disease 2019 (COVID-19) has infected millions of individuals around the globe. Forecasting the COVID-19 severity is essential, and various biomarkers could be used to evaluate it. The current study was therefore aimed to evaluate the serum pro-calcitonin (PCT) level as a biomarker for bacterial co-infection and disease severity in COVID-19 patients. A total of 430 COVID-19 positive individuals were examined, in which 332 (77.2%) were male individuals while 98 (22.8%) were female individuals. Among the examined samples, 281 were classified as moderate (PCT value 0.07 ± 0.06 ng/mL), 95 were severe (PCT value 0.5 ± 0.4 ng/mL), and 54 were classified as critical (PCT value > 1 ng/mL) individuals. The increase in the total serum level of PCT was observed with the severity of the disease (p < 0.05). The statistical analysis represented no association of PCT value with gender (p 0.9650) while revealed a significant association (p < 0.001) with the age and PCT value in COVID-19 patients. It can be concluded that the serial PCT measurement could determine the prognosis of the disease and the presence of bacterial co-infection in COVID-19 patients. Further exploration of the topic is needed to evaluate the effect of different therapies on the PCT level and to prescribe specific treatment options for coinfection.

## Introduction

In the Twenty-First century, emerging viral infections are among the greatest challenges in the public health sector [1]. Among them, most of the viruses jump from other species of animals to humans, called zoonotic viruses [2]. Coronaviruses, by crossing specie-barrier, have infected the human population for the third time in the current century [3]. In late December 2019, numerous cases of a novel severe respiratory disease that were characterized by fever, dry cough, shortness of breath, chills, myalgia, nasal congestion, and muscle pain were described in Wuhan city, Hubei Province, China [4]. On 30th December 2019, the centre for disease control (CDC) China reported that pneumonia has an unknown aetiology [5]. On 7th January, the causative agent of pneumonia was isolated, and the whole-genome sequence was released in early January 2020. It was recognized that the deadly pneumonia was caused by a virus that belongs to the coronaviridae family in the Nidovirales order of coronaviruses [4,[6], [7], [8]]. The virus was initially named as 2019 novel coronavirus (2019-nCoV). Later, the World Health Organization (WHO) named it severe acute respiratory syndrome coronavirus 2 (SARS-CoV-2) and the disease it caused as coronavirus diseases 2019 (COVID-19) on February 11, 2020 [9]. Due to its high contagion and transmission rate, COVID-19 spread throughout the globe [10]. WHO first declared it a public health emergency of international concern and then as a pandemic [11]. As of 31st March 2021, the WHO reported 127.3 million confirmed cases with 2.7 million mortalities around the globe [12].

The forecasting of COVID-19 severity is essential in the current pandemic. The disease severity could be evaluated by various laboratory markers, including serum pro-calcitonin, C-creative protein and D-dimers [13]. Procalcitonin (PCT) is a precursor of hormone calcitonin [14]. In normal individuals, it is produced by the parafollicular cells of the thyroid. However, in cases of different bacterial infections, the mesodermal cells in various tissues such as the lung, intestine, liver, kidney, and fat tissues are responsible for the production of PCT [15]. Serum PCT has been considered as one of the main biomarkers of bacterial infection, which could be incorporated into decision-supporting and prognostic algorithms for the management of lower respiratory tract infections [16]. PCT testing has been suggested in critical patients because raised serum biomarker concentrations may reveal a superimposed bacterial co-infection, which, in turn, supplements disease severity and possibly drives systemic sepsis, also allowing a more targeted use of antimicrobials and promoting antibiotic stewardship even in COVID-19 [37]. Besides, PCT could be helpful in the identification of critical patients at high risk of organ injury. Recently, several findings revealed that a greater level of serum PCT is associated with COVID-19 severity [13]. It has been reported that the mean serum PCT level is much higher in critical and severe patients of COVID-19 as compared to moderate COVID-19 patients [17]. PCT has also been included in the recommended laboratory work-up for the better prediction of prognosis and to improve the diagnosis to differentiate the moderate, severe, and critical patients of COVID-19 [18].

Different studies revealed a high prevalence of Bacterial co-infection during COVID-19 pneumonia as an early bacterial co-infection primarily associated with *S aureus*. Recently, bacterial respiratory infection in critically ill COVID-19 patients was estimated between 14 and 28% [[38], [39]–40]. The use of antibacterial has also been markedly increased in the face of SARS-COV-2 infection, posing antimicrobial resistance. PCT testing could offer a predictory value of bacterial co-infection and be a valuable marker for assessing disease severity. As demonstrated by the PRORATA trial, in ICU patients with lower respiratory tract infections, PCT values < 0.25 μg/L indicate bacterial aetiology as very unlikely, while values ≥ 1.0 μg/L indicate a high probability for this aetiology. Attending physicians should be alert of the significantly raised proportion of bacterial co-infections, specifically *S aureus*, during COVID-19 pneumonia. However, previous trial results and specific guidelines did not support antibiotics prescriptions for patients with PCT values ≤ 0.1 ng/mL. In comparison, patients with PCT values ≥ 0.5 ng/mL are strong candidates for antibiotics administration. The aim of the present study was to investigate the serum PCT level of COVID-19 patients and to determine the role of PCT as an indicator of COVID-19 severity.

## Materials and methods

### Study population and sample collection

The current study was conducted at Al-Habib Clinical Laboratory Kohat, Pakistan. A total of 430 COVID-19 positive patients were examined in this study. Among the total individuals, 332 (77.2%) were male, while 98 (22.8%) were female. A written consent was taken from all the individuals. About 3 ml blood sample was obtained from each individual in a gel vacutainer tube. The relevant demographic information like age and gender were also recorded.

### Inclusion criteria

All those individuals who were tested positive for the SARS-CoV-2 according to the WHO and CDC guidelines for the detection and diagnosis of COVID-19 were included in this study.

### Sample processing

The collected samples were centrifuged at 8000 rpm for 10 minutes to obtain a clear supernatant serum. The samples were analysed through Finecare™ PCT Rapid Test. The net result display by the Finecare™ FIA Meter is in the unit of ng/mL as XXX ng/mL and was analysed later.

### Definitions

The COVID-19 is categorized into mild, moderate, severe and critical pneumonia by the New Coronavirus Pneumonia Prevention and Control Program (6th edition). In mild pneumonia, mild clinical symptoms with normal chest imaging could be observed. Moderate pneumonia is classified as the presence of abnormal chest imaging along with clinical symptoms. Severe pneumonia is classified when a patient fulfils one of the three criteria, including increased respiration rate (more than 30 breaths per minute), oxygen saturation in resting state (less than 93%), and/or the ratio of oxygen partial pressure to a fraction of inspired oxygen (less than 300 mmHg), while the patients are diagnosed with critical pneumonia when the disease represents rapid progression toward shock, respiratory failure (need of ventilator), and/or organ failure. The patients in critical pneumonia conditions need to be admitted to the intensive care unit (ICU) for special monitoring. Bacterial infections were defined on the basis of increased white blood cells (WBCs) counts and the increased level of PCT.

### Statistical analysis

The statistical analysis of the data was performed using Statistical Package for the Social Sciences (SPPS). The qualitative analysis of the data was performed by the Chi-square test. The p < 0.05 was considered significant. The correlation between different variables was examined.

## Results

**Fig. 1:**
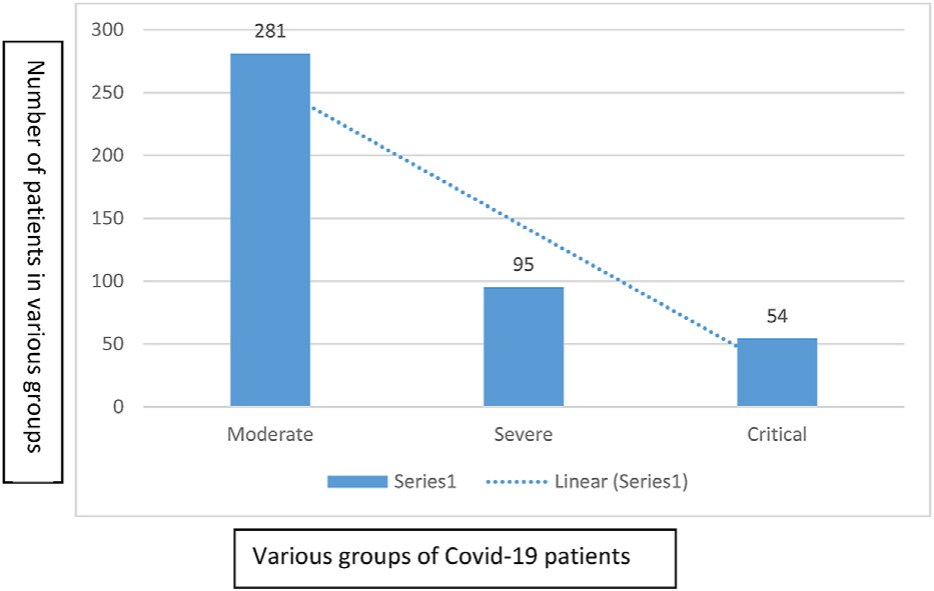
Different groups based on COVID-19 severity along with the number of cases. Among all patients, 281 were moderate, 95 were severe, and 54 were critically ill patients.

**Fig. 2:**
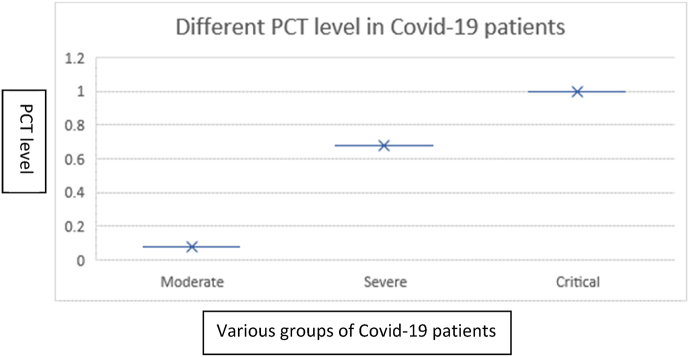
Different PCT levels in various groups of COVID-19 patients. The critically ill disease group was the highest level of PCT level >1 (1.2 ± 0.2-5.5 ± 0.5) compared to severe and moderate groups of patients. Chi-square test and one way ANOVA were applied to determine the association. Significance of p < 0.05 was considered at 95% confidence interval.

A total of 430 COVID-19 positive individuals were examined in this study. The COVID-19 patients were previously screened through RT-PCR. Among the total individuals (n = 430), 332 were male individuals while 98 were female individuals. Among the examined samples, 281 (65.3%) were moderate, 95 (22%) were severe, and 54 (12.5%) were critical individuals (Fig. 1 and Table 1). In our study, the overall mean age observed was 45.8 years. The mean age in the moderate class was 45 ± 7, severe was 57 ± 8, and critical was 70 ± 5 (Table 1). The increase in the total serum level of PCT was observed with the severity of the disease (p < 0.05). The mean PCT level for moderate group was 0.07 ± 0.06 ng/mL, for severe group was 0.5 ± 0.4 ng/mL, and was >1 ng/mL (1.2 ± 0.2-5.5 ± 0.5) for critical group (Fig. 2 and Table 1). The statistical analysis revealed that there was no association of PCT value and gender (p 0.9650) while there was a significant association (p < 0.001) of age and PCT value in COVID-19 patients.

**Table 1:** Different groups of COVID-19 patients along with their age and PCT level

Group	No. of patients	Mean age	Mean PCT level
Moderate	281	45 ± 7	0.07 ± 0.06
Severe	95	57 ± 8	0.5 ± 0.4
Critical	54	70 ± 5	>1 (1.2 ± 0.2-5.5 ± 0.5)

## Discussion

PCT is a precursor of calcitonin made of about 116 amino acids and is produced by parafollicular cells of the thyroids [19,20]. PCT could also be synthesized during bacterial infection in other tissues, including lungs, intestine, kidney, liver [21]. In a non-infected person, the average blood PCT level is 0.033 ng/ml, and it cannot be detected by the commonly used methods in clinical laboratories. Hence, blood PCT levels below the assay’s detection limit serve as an excellent negative predictor for bacterial community-acquired respiratory and nosocomial infection. The production of PCT during bacterial infection is mediated by tumour necrosis factor-alpha and interleukin 6, which increases during infection [16]. Comparing to other molecules produced during infection, PCT has a high value for bacterial infections [20,22,23]. In previous studies, the PCT value has been observed to be elevated and are directly related to the COVID-19 severity [23,24]. Other studies have revealed that greater PCT concentrations increase the risk of severity in diseases [25].

Previously researchers reported that the PCT level were approximately four times higher in the severe group than in the moderate group and approximately eight times higher in the critical group than in the moderate group [17]. In our study, we also observed similar findings, and we observed that the elevation in PCT level increases with the increase in age of the patients. The fluctuation in PCT level could be associated with the prognosis of the diseases [26]. In a similar study, the COVID-19 patients were categorized into moderate, severe, and critical classes based on clinical characteristics, including PCT values [17]. It has been previously observed that most of the COVID-19 patients have very low PCT level, while in admissions to the hospital, markers of inflammation like C-creative proteins (CRP) and biochemical and other biomarkers for the bacterial infections, however, indicated infection in the lungs. Most COVID-19 patients have very low PCT levels (moderate) at the time of admission, but elevated markers of inflammation such as white blood count (WBC) and C-reactive protein (CRP) indicate that they have inflammation in the lungs [[27], [28], [29]]. However, the PCT levels increase during the course of the disease, which could be linked to the presence of bacterial infections. The bacterial co-infection with the viral infection could result in the development of secondary bacterial pneumonia in these patients [30]. Therefore, when the bacterial co-infection is confirmed, the patients should be treated with antibiotics [31]. However, bacterial co-infections could increase prognosis and mortality risk if early antibiotic treatment is not initiated [32]. The co-infection could also worsen the already overburdened health systems and increases the risk of other outbreaks [[33], [34], [35]].

Therefore, the examination of PCT as a biomarker is essential in COVID-19 patients. For the identification of bacterial infections, PCT is a better biomarker as it has specificity towards bacterial infections. PCT levels alone could not prove the presence of co-infection, and therefore, pathogens derived markers like sputum culture and PCR need to be performed. PCT as effective biomarkers and prognostic tool for lungs infection and other respiratory tract infections has been reported previously. Therefore, the analysis of PCT level in COVID-19 patients is very important, and several studies have reported its significant prognostics implication and detection of bacterial co-infections [36]. In addition to other prognostic tools as infection bio-markers for monitoring of COVID-19 patients, PCT could also be used as an important biomarker. Our results demonstrated that the serial PCT measurement could predict the prognosis of COVID-19 patients. Although PCT could be less affected by steroid use, however, more exploration of the topic is needed to better understand the effect of different treatments and their influence on the PCT level. Moreover, the primary application of PCT in COVID-19 is its ability to exclude a bacterial co-infection associated with a negative p > 90% when a rule-out cut-off (<0.25 μg/L) is applied [41].

## Conclusion

From the current study, it could be concluded that the serum PCT measurement could determine the prognosis of the disease and the presence of bacterial co-infection in COVID-19 patients. By comparing the PCT levels in severe patients, we found statistically significant differences and demonstrated that PCT might determine the severity of patients infected with SARS-CoV-2. Thus, the serum PCT measurement could evaluate the prognosis of the disease and the presence of bacterial co-infection in some COVID-19 patients. However, the role serum procalcitonin (PCT) measurement plays in laboratory practice have been primarily argued by discussing the accessibility of robust data and the lack of scientific evidence affecting its clinical use. Moreover, it is not a reliable marker within 48 h of admission to determine bacterial co-infection. Further studies are needed to evaluate the effect of various drug treatments on the PCT level, the process by which the PCT is synthesized and released and to prescribe specific treatment for co-infection.

## Transparency declaration

The authors declare that they have no known competing financial interests or personal relationships that could have appeared to influence the work reported in this paper.

## Funding

Not available.

## Ethical statement

This study has been approved by the departmental ethics committee.

## Authors contribution

AW and MD contributed to conceptualization and methodology. AW and MD, and AK contributed to data curation, writing and original draft preparation. LY, MN, and AB contributed to visualization and investigation. MA supervised the study and contributed to writing, reviewing and editing the paper.
